# A Miniature Fabry–Pérot Fiber Interference Sensor Based on Polyvinyl Chloride Membrane for Acoustic Pressure Sensing in Mid–High-Frequency Band

**DOI:** 10.3390/ma14247605

**Published:** 2021-12-10

**Authors:** Qingkai Yao, Xing Guo, Linfang Xie, Li Sun, Fapeng Yu, Xian Zhao

**Affiliations:** 1 Center for Optics Research and Engineering, State Key Laboratory of Crystal Materials, Shandong University, Jinan 250100, China; qingkyao@163.com (Q.Y.); 201912580@mail.sdu.edu.cn (X.G.); 202012900@mail.sdu.edu.cn (L.X.); sdusunli@sdu.edu.cn (L.S.); 2 Shandong Original Crystal Technology Co., Ltd., Jinan 250100, China

**Keywords:** acoustic sensing, Fabry–Pérot, polyvinyl chloride, optical fiber sensor

## Abstract

In this paper, a Fabry–Pérot interference fiber sensor was fabricated by using a Polyvinyl chloride membrane (20 μm in thickness) attached at the end of a ferrule with an inner diameter of 1.1 mm. In consideration of the vibration response of the membrane, the feature of the first-order natural frequency of membrane was analyzed by COMSOL Multiphysics. The acoustic sensing performance of the Fabry–Pérot fiber interference sensor was studied in air. The results reveal that the sensor possessed good acoustic pressure sensitivity, in the order of 33.26 mV/Pa. In addition, the noise-limited minimum detectable pressure level was determined to be 58.9 μPa/Hz^1/2^ and the pressure-induced deflection obtained was 105 nm/Pa at the frequency of 1 kHz. The response of the sensor was approximately consistent with the reference sensor from 1 to 7 kHz. All these results support that the fabricated Fabry–Pérot fiber interference sensor may be applied for ultra-sensitive pressure sensing applications.

## 1. Introduction

Ultra-sensitive sensing technology has important applications in many fields, such as acoustic wave acquisition, acoustic source location, acoustic imaging, fault voiceprint detection and early warning. Besides, it has admirable application prospects in photoacoustic spectrum (PAS) gas detection. Ultra-sensitive gas detection technologies are desired in the fields of gas leakage, ultra-high voltage (UHV) partial discharge, industrial fault detection and medical diagnosis. Therefore, the development of the miniaturized and flexible distributed sensor has become an academic hotspot.

Research on fiber optic Fabry–Pérot interference (FPI) sensors have a long tradition for obtaining static and dynamic pressure measurements due to outstanding merits, such as ultra-sensitivity and suitability for use in hostile environments and multi-channel perspectives for meeting the demand of many civil and military fields [1,2].

Compared with other optical fiber interferometers, such as Mach–Zehnder and Michelson interferometers, FPI sensors are almost unaffected by fiber length [3,4,5]. The sensor, which is composed of a fiber core and a pressure-sensitive diaphragm, is commonly established directly on the end face of the cleaved single-mode fiber [6,7]. Such sensors are referred to as extrinsic Fabry–Pérot interferometric sensors (EFPIs) [8]. Commonly, a columniform ferrule, small in size, is applied to form an interference cavity. Therefore, the extreme micro-size and flexible distribution allows them to be applied to large-scale distributed sound field detection systems, for instance oil and gas pipeline leakage monitoring, high-precision PAS detection, seismic wave monitoring and anti-submarine monitoring [9,10]. Meanwhile, with the uninterrupted development of power systems and the increasing demand for power supply reliability in civil fields, the maintenance mode of large power equipment has been gradually transformed from regular maintenance to condition monitoring. In these oil and gas monitoring technologies, PAS has plenty of outstanding aspects, such as satisfying gas selectivity and low cross-interference [10]. However, most of the acoustic sensors currently applied in PAS systems are commercial microphones, which are not only expensive but also bulky [11]. Therefore, the micro-sized FPI sensors become a good choice, because they are uncomplicated to combine with PAS systems, they hardly disturb the acoustic field distribution of the PA resonant cavity in the form of first-order resonance and they allow a higher detection limit to be achieved [4]. The operating frequency, which is generally below 4 kHz in first-order longitudinal resonance PAS systems, is determined by the size of the resonant cavity [12,13].

Among lots of EFPI sensors, the response characteristics of silicon, polymer film, silver film and graphene membrane graphene diaphragm are more satisfactory due to their own excellent mechanical properties. According to relevant literature, fewer-layer graphene films with a diameter of 125 μm and a thickness of 100 nm achieve a pressure sensitivity of 1.1 nm/Pa [14]. However, they only focus on obtaining a higher first-order resonance frequency to obtain a wider frequency response range and are not optimized for the sensors required for a resonant PAS. The key factor which decides the dynamic pressure performance of the FPI sensor is the mechanical parameter and size of the applied membrane. According to the theory of film resonance, larger-sized membranes are more suitable for fabricating FPI sensors for obtaining a satisfactory lower-frequency response [15]. However, for films with a thickness of several microns, the larger diameter increases the difficulty of the film-transfer process. Meanwhile, the current immature growth process of high-quality graphene makes it prone to fracture in the state of large size [16]. Therefore, it is very necessary to adopt suitable materials for membranes with a larger diameter to obtain a lower detectable limit.

In this study, an FPI sensor was designed and fabricated using a Polyvinyl chloride (PVC) membrane with a 1.1 mm diameter. The natural frequency of the membrane’s model was simulated and studied using COMSOL Multiphysics. The output characteristics of the sensor indicated that the sensitivity of the membrane can reach 33.2 mV/Pa; furthermore, the noise-limited minimum detectable pressure (MDP) level was 58.9 μPa/Hz^1/2^ at a sonic frequency of 1 kHz. The frequency response of the sensor was approximately consistent with the reference sensor below 7 kHz.

## 2. FPI Pressure Sensor Design and Simulation

### 2.1. FPI Pressure Sensor Design and Fabrication

**Figure 1:**
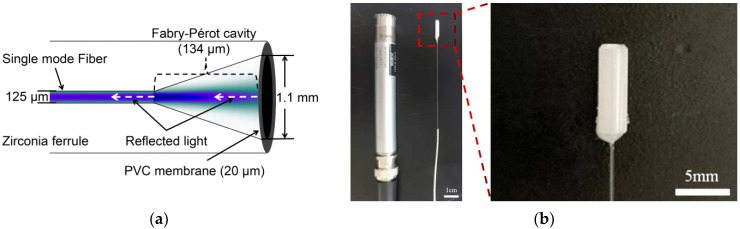
(**a**) Diagrammatic sketch. (**b**) Comparison of the manufactured FPI sensor and the reference microphone.

In this study, a PVC diaphragm was adhered to a zirconia substrate with an inner diameter of 1.1 mm using a temperature-adjustable plastic welder for making the PVC diaphragm thoroughly stick to the end face of the substrate. Because the deflection is inversely proportional to Young’s modulus, it is necessary to decrease the Young’s modulus of the PVC diaphragm from the initial 3 GPa to 6 MPa on account of plastication [17]. Therefore, we adopted 280 °C and 30 s as the heating conditions for the welding process. From beginning to end, the zirconia substrate was kept perpendicular to the air outlet to avoid denaturation and deflection of the PVC membrane. Deflection caused by the manufacturing process may lessen the sensitivity of the FPI sensor [18]. Figure 1a displays the diagrammatic sketch of the sensor. The cleaved fiber was inserted into the ceramic ferrule and formed an interference chamber with the PVC membrane. Figure 1b is the physical image compared with the reference sensor. Epoxy was used to fix the optical fiber to the zirconia ferrule.

**Figure 2:**
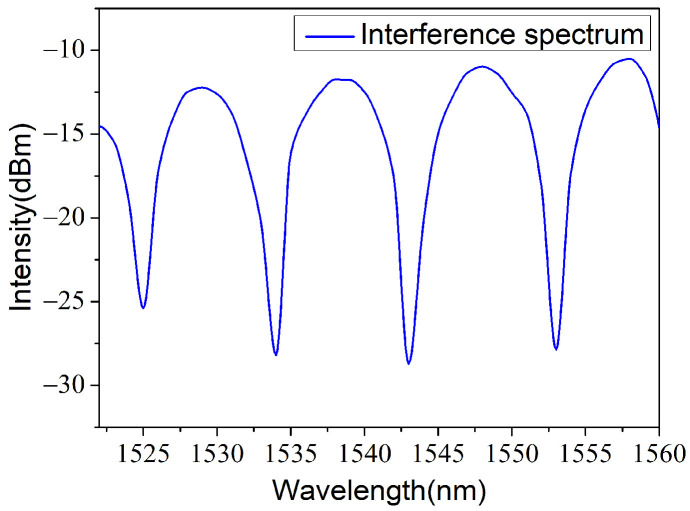
Measured reflection spectra of the FPI.

The coupling efficiency of the reflected light decreases as the cavity length increases because of the divergence angle of the light emitted from the fiber. For obtaining better interference spectrum sharpness, it is necessary to control the relative position of the optical fiber and ceramic core more finely. Accordingly, the optical fiber was fixed to a high-resolution translation stage in order to precisely adjust the separation of the interference cavity. The width of the partition between the membrane and cleaved end was determined by

(1) $${L=\lambda }_{1}\lambda_{2}/2(\lambda_{1}-\lambda_{2}),$$

where $\lambda_{1}$ and $\lambda_{2}$ are wavelengths of two adjoining peak/dip in the systematic spectrum [19,20]. As shown in Figure 2, a finely represented interference spectrum was measured. The fringe visibility was approximately 20 dB and the free spectral range was 9 nm. Calculated by Equation (1), the distance between fiber end face and PVC film obtained was approximately 134 μm.

### 2.2. Natural Frequency Analysis of Pressure-Sensitive Membrane

**Figure 3:**
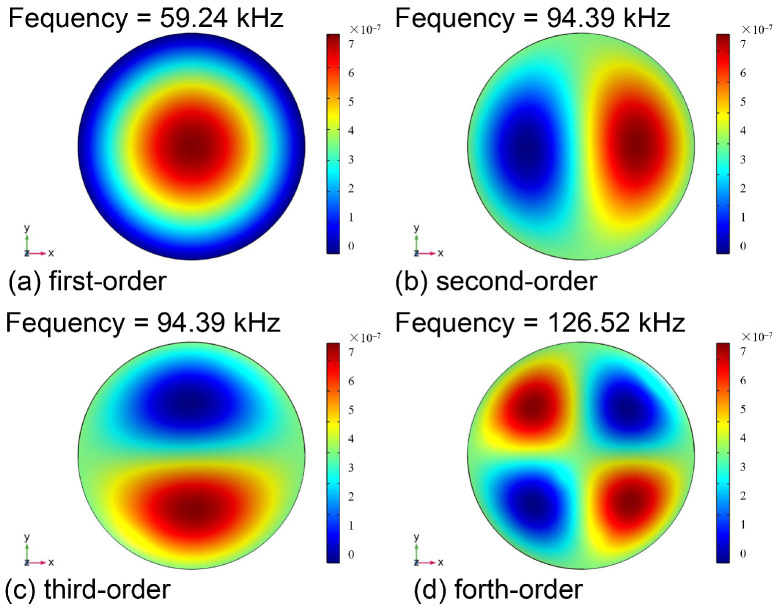
Simulation results of the first four natural frequencies.

To obtain accurate acoustic pressure, the operating frequency of the circular PVC membrane should be much lower than the resonant frequency [15]. The analysis of the membrane’s natural frequency is critical, particularly to guarantee the output characteristics of the sensor at different frequencies. Assuming the PVC diaphragm is tightly bound to the zirconia substrate, the first-order natural frequency $f_{00}$ can be indicated by the following formula [21]:

(2) $$f_{00\;}=\frac{10.21t}{{2\pi r}^{2}}\sqrt{\frac{E}{12\rho \left(1-\upsilon^{2}\right)}},$$

where mass density ρ was $1.27\times 10^{3}\;\mathrm{kg}/m^{3}$, thickness t was 20 μm, radius r was 1.1 mm and Poisson’s ratio ν was 0.4 for the PVC diaphragm used in this study. Based on the above parameters, $f_{00}$ was calculated and determined to be 61.5 kHz. To further verify the resonance frequency of the membrane, COMSOL Multiphysics was adopted to establish and simulate the three-dimensional cylinder model. The simulation results of the first four resonant frequencies are illustrated in Figure 3. It was found that the second-order and third-order resonant frequencies had two poles with maximum deflection and, concurrently, the fourth-order resonant frequencies had four extreme deformation points, which is not consistent with the way the membrane works. According to the simulated results, the first-order resonance frequency of the PVC membrane with 1.1 mm in diameter had the value of 59.24 kHz, as recorded. This was consistent with the one calculated (61.5 kHz) with Equation (2). The required operating frequency in a first-order longitudinal PA resonance model is usually below 4 kHz. This frequency is much lower than the natural frequency of the PVC diaphragm adopted in the study.

We supposed that the circular disk film model has fixed edges; then, the formula to describe membrane deflection changes with acoustic excitation and can be written as [18]

(3) $$\Delta L=\frac{3(1-\nu^{2}{)D}^{4}}{{256\mathrm{Et}}^{3}}\Delta P,$$

where D, t, ν and E are the diameter (1.1 mm), thickness (20 μm), Poisson’s ratio (0.38) and Young’s modulus (6 MPa) of the membrane, respectively. As expressed in Equation (3), the greater the deflection caused by acoustic pressure, the smaller the minimum detectable quantity that can be achieved. The theoretical deflection of the PVC film with a diameter of 1.1 mm and a thickness of 20 μm exploited in the experiment was about 100 times than the deflection of a graphene film with 125 μm in diameter and 100 nm in thickness [14].

In addition, the actual deflection of the PVC membrane was calculated. The distinction of the interference spectrum is decided by the reflectivities of the two interfaces forming the interference cavity. One reflectivity, R1, was evaluated to be 0.02 before the unclad fiber was inserted into the ferrule. Correspondingly, the reflectance of the PVC membrane R2 was about 0.4%, after fitting the data in Figure 2. In order to verify the reliability of this result, the theoretical value based on Fresnel’s law of refraction and reflection was estimated. Here, the refractive index of the PVC membrane was about 1.52. The calculated refractive index was approximately 0.43%. Further, the deflection of the diaphragm may be caused by the process of sensor manufacturing and might be the main reason for which the measured reflectivity was smaller than the theoretical one. As shown in Figure 1a, the light coming out of the fiber has an angle of divergence that would be declined by the reflection efficiency of light. Under the approximation of two-beam interference, the relationship between the voltage of the output AC component V and the deflection in the center of the film ΔL can be written as the following formula [19]:

(4) $$V=R\cdot I_{0}\cdot S_{\max}\cdot \Delta L,$$

where R (~940 mV/μW at 1500 nm) is the InGaAs detector sensitivity and $I_{0}$ (~2.1 μW) is the total light power. In addition, the slope coefficient $S_{\max}$ can be written as

(5) $$S_{\max}=2\sqrt{R_{1}R_{2}}\cdot \frac{4\pi n}{\lambda },$$

According to Equation (4), the relationship between the deflection of the PVC membrane and acoustic pressure was determined to be approximately 105 nm/Pa. This result is about 95 times the one reported in a previous study [14] and it is approximate to the value calculated by using Equation (3). Therefore, based on the above analysis, a larger-sized PVC film is more suitable for lower-frequency sonic investigations, compared to the membrane with a smaller diameter and higher Young’s modulus, because it has a lower first-order resonance frequency, as shown in the simulation results in Figure 3; further, a larger deflection can be obtained at the same sound pressure level.

## 3. Experimental Section

**Figure 4:**
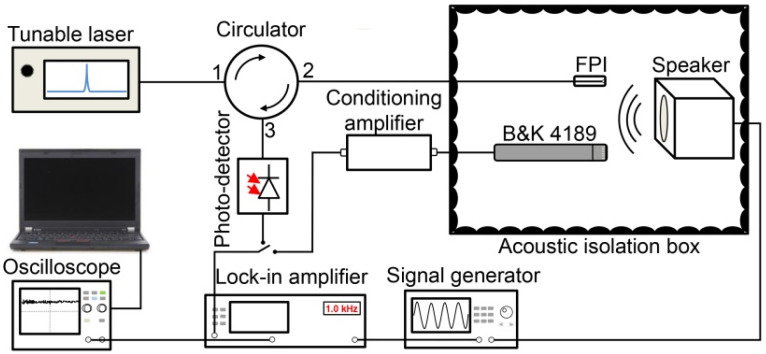
Schematic diagram of pressure test experimental setup.

To verify the sensor’s potential in detecting mid–high-frequency acoustic waves, the testing system was designed; a diagrammatic representation of the experimental system is delineated in Figure 4. The fabricated sensor and purchasable microphone (B&K4189) were established in a position symmetrical to the speaker, which was driven by a function signal generator (AFG1062, Tektronix, Beaverton, OR, USA), where B&K4189, accompanying a sensitivity of 50 mV/Pa, was used as a reference microphone to calibrate the standard acoustic pressure. To eliminate environmental noise, they were all equipped inside a soundproof box. The tunable wavelength laser output was irradiated to the PVC membrane through the circulator and the wavelength was adjusted to the quadrature (Q) point of an interference spectrum to improve the electrical response [19]. An optical fiber circulator was operated to separate the light output by the laser from the light reflected by the PVC membrane. A photo-detector (GDT-D002N, Daheng Optics, Beijing, China) was applied to receive the intensity of optical energy of the output of the circulator. Then, the dual-channel electrical signal output was delivered to a phase-locked amplifier (SR830, Stanford Research Systems, Sunnyvale, CA, USA) or oscilloscope (MDO3022, Tektronix). The data acquisition card (GPIB-USB-HS, National Instruments, Austin, TX, USA) was connected to the lock-in amplifier to obtain the digital signal under a low-pressure level. In addition, the waveform of the signal was obtained through PC control of the oscilloscope using USBTMC (USB Test and Measurement Class) protocol.

## 4. Results and Discussion

**Figure 5:**
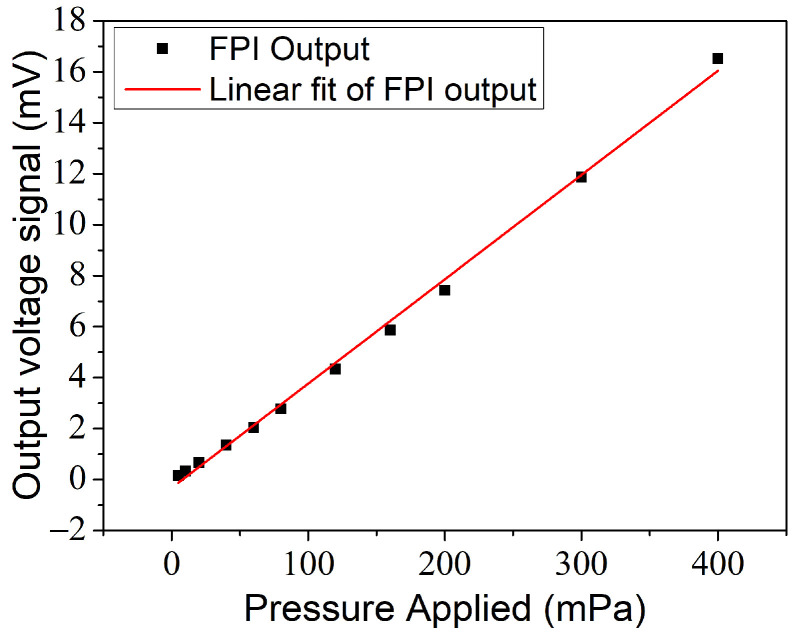
The output electrical signal under different acoustic pressure levels at 1 kHz.

Considering the response characteristics of the sensor, we investigated the output of the FPI under different sonic pressure levels. Labview was exploited to acquire the time-domain signal and perform low-pass filtering. After eliminating the DC component, Figure 5 shows the output voltage peak-to-peak value of the FPI obtained by the photo-detector at the frequency of 1 kHz. When the sound pressure value was below 400 mPa, it showed a tolerable linear relationship. The coefficient of determination (R-square) was about 0.9967. Though the sensitivity of the manufactured FPI approach was 33.26 mV/Pa, which is not as high as that of B&K4189, the advantages of the sensor in terms of size and long-distance transmission make it more flexible in distributed applications.

**Figure 6:**
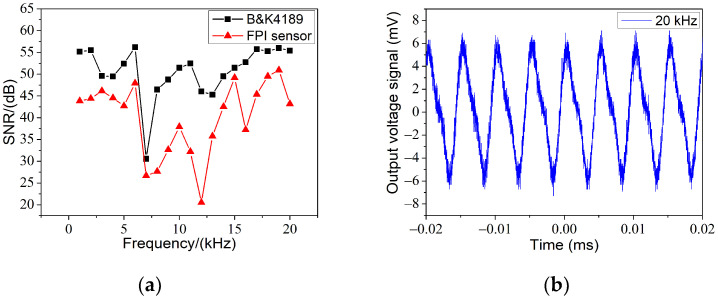
(**a**) Frequency spectrum of the FPI from 1 to 20 kHz at an acoustic pressure level of 300 mPa. (**b**) Dynamic response of FPI at 20 kHz.

We noticed that the signal-to-noise ratio (SNR) of the fabricated FPI sensor and the B&K4189 sensor were consistent, in the range 1–7 kHz, as shown in Figure 6a. Although the trends for the FPI sensor and B&K4189 sensor were similar, there was precarious deviation in the range of 10–20 kHz and the largest deviation occurred at 12 kHz. Two reasons are believed to be accountable for this phenomenon. Firstly, the frequency band reached one-third of the natural frequency of the film, which made it difficult for the sensor to capture a real signal. Secondly, the reference sensor was a free-field microphone, while the FPI sensor was not optimized for a free field. As exhibited in Figure 6b, the electric signal obtained was severely distorted at 20 kHz. Therefore, in order to improve the response of acoustic waves in a wider frequency range, the film of the sensor should be optimized. Film materials with higher Young’s modulus, smaller diameter and thinner thickness would be an appropriate choice.

**Figure 7:**
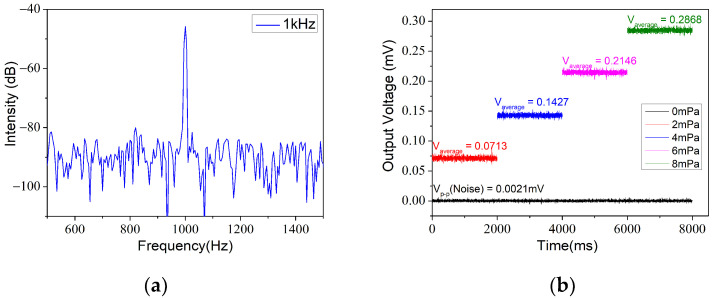
(**a**) Power spectrum of signal at 1 kHz. (**b**) Output of lock-in amplifier at low acoustic pressure level at 1 kHz.

Noise is an essential qualification during the design of a detector. There are many methods to define or analyze noise [22]. In this paper, we calculated two consequences of detectable limit. First at all, a 30 Hz resolution bandwidth power spectrum acquired by the digital oscilloscope at an acoustic pressure level of 300 mPa is shown in Figure 7a. A low MDP can be expressed as

(6) $$\mathrm{MDP}=\frac{P}{\sqrt{10^{\mathrm{SNR}/10}}\times \sqrt{\mathrm{RBW}}},$$

where P is the acoustic pressure and RBW is the resolution bandwidth. The noise level was approximately −90 dB and the SNR was roughly estimated to be 48 dB; then, the result of the calculation of the noise-limited MDP level approach was estimated to be 217 μPa/Hz^1/2^ under a 1 kHz excitation frequency.

Furthermore, employing advanced approaches such as lock-in technology may allow faint effective signals to be detected. Currently, it is one of the most widely used weak-signal detection techniques in the laboratory [13,14,18,19,20]. From this point of view, a lock-in amplifier was used to detect the electrical signal output characteristics at low sound pressure levels at 1 kHz. Figure 7b shows the voltage value obtained using a phase-locked amplifier for different applied pressure levels at 1 kHz. The integration time of the lock-in amplifier was turned to 100 ms and the slope was 18 dB/oct. It can be seen from the figure that, at 0 mPa and 2 mPa, the output amplitude was 0.021 mV and 0.0713 mV, respectively. Correspondingly, the noise-limited MDP level was about 58.9 μPa/Hz^1/2^. In summary, we list several characteristics of the FPI sensor compared with the reference sensor in Table 1. It should be noted that a better detection limit can be obtained by further extending the integration duration of the lock-in amplifier. Although not as good as the performance of B&K4189, the FPI still has its unique advantages, such as minuscule size, long-distance measurement, EMI immunity and flexibility, etc.

**Figure 8:**
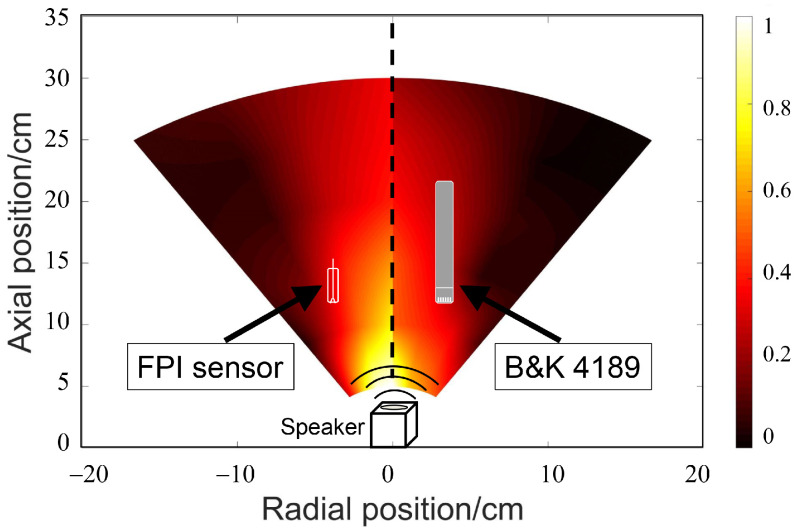
Normalized acoustic response of the FPI sensor and B&K4189 in fan-shaped region.

In order to investigate the directivity of the manufactured sensor, the FPI sensor and B&K4189 were placed in the fan-shaped area of the acoustic field and the response of the sensors was recorded. After expanding the data by interpolation, the normalized data for assorted alignment angles were projected into the fan-shaped area, as shown in Figure 8. The composite image shows the FPI sensor’s (left) and B&K4189’s (right) normalized acoustic sensitivities. It can be concluded, from the comparison with the reference sensor, that the FPI sensor showed satisfactory omnidirectionality.

## 5. Conclusions

A miniature FPI sensor, adopting PVC as a pressure-sensitive film, was manufactured based on a cylindrical ferrule with a length of 10.5 mm and a diameter of 2.4 mm in the interest of acoustic pressure sensing in the mid–high-frequency band. The pressure-sensitive film mechanical modeling and simulation by exploiting COMSOL Multiphysics indicated that the first-order natural frequency was about 61.5 kHz, which can satisfy the demand of sonic detection in the mid–high-frequency band. Furthermore, the electrical signal sensitivity of the sensor obtained was approximately 33.23 mV/Pa. Moreover, the noise-limited MDP level achieved was as high as 58.9 μPa/Hz^1/2^ at 1 kHz. Although sinusoidal acoustic waves showed significant distortion near 20 kHz, the FPI sensor could impeccably express the acoustic characteristics under 7 kHz. The minuscule size, long-distance measurement, EMI immunity and flexibility of the PVC-based FPI show the advantages of this sensor for acoustic pressure sensing application.

## Figures and Tables

**Table 1:** The characteristics of the FPI sensor compared with B&K4189.

Sensor	Size	MDP	Sensitivity
FPI	Φ2.4 mm × 10.5 mm	58.9 μPa/Hz^1/2^@1 kHz	33.26 mV/Pa
B&K4189	Φ1.2 cm × 9.5 cm	8 μPa/Hz^1/2^	50 mV/Pa

## Data Availability

The data presented in this study are available on request from the corresponding author.
